# Evaluation of skin of color content in *Skin Research and Technology*

**DOI:** 10.1111/srt.13105

**Published:** 2022-01-19

**Authors:** Mary Sun, Britney N. Wilson, Rebecca Z. Zhou, Dedee F. Murrell, Jenny E. Murase

**Affiliations:** ^1^ Icahn School of Medicine at Mount Sinai New York New York USA; ^2^ School of Medicine Rutgers New Jersey Medical School Newark New Jersey USA; ^3^ The George Institute of Global Health Newtown New South Wales Australia; ^4^ Sydney Faculty of Medicine University of New South Wales Sydney New South Wales Australia; ^5^ Department of Dermatology University of California, San Francisco San Francisco California USA; ^6^ Department of Dermatology Palo Alto Foundation Medical Group Mountain View California USA

To the Editor,

Dermatology is among the least diverse medical specialties, and many dermatologists report inadequate training regarding diseases in skin of color (SOC).^1^ Assessment of educational dermatological resources, including textbooks and continuing medical education (CME) events, reveals a lack of emphasis on and overall underrepresentation of disease patterns in SOC.^2^ Limited exposure to disease presentation in darker skin tones may result in delayed or missed diagnoses and subsequent suboptimal management of conditions in SOC populations. Since its conception in 1995, *Skin Research and Technology* has published many articles relevant to SOC, including those that address and compare differences in skin, eye, and hair properties across various ethnicities.^3^, ^4^, ^5^, ^6^, ^7^, ^8^, ^9^ Assessment of diversity and SOC‐related content in high‐impact academic dermatology journals including *Skin Research and Technology is* of great interest, as academic literature serves as an important educational resource for treating patients of color. This study developed and applied prespecified criteria to evaluate the representation of SOC‐relevant publications in recent, peer‐reviewed dermatology journals.

We reviewed articles published in 52 dermatology journals from January 2018 to October 2020, using prespecified criteria that assess SOC in dermatologic literature (Table 1).^10^ Journals were selected based on impact factor and Scopus ratings and were classified as either SOC (journals that emphasize countries with population majorities of Fitzpatrick skin type III or higher) or non‐SOC, and scientific or clinical. The 26 journals with the highest Scopus CiteScores received a “High” CiteScore designation, and the remaining 25 journals were labeled as “Low.” Analyses were conducted in R software, version 4.0.2.

**TABLE 1:** Pre‐specified criteria for assessment of skin of color relevance in dermatological literature

Tier	Criteria
**1A**	Title addresses SOC, skin type, race, or ethnicity
**1B**	Title addresses country or continent with population majorities of Fitzpatrick skin type III‐VI
**1C**	Title addresses socioeconomic and health disparities relevant to under‐represented SOC populations
**1D**	Title addresses issues regarding diversity and inclusion in field of dermatology
**1E**	Case reports presenting a SOC patient in a non‐SOC country
**2**	Title addresses pigmentary skin or hair diseases particularly relevant to Fitzpatrick skin types III‐VI

Of the 52 peer‐reviewed dermatology journals included in our study, *Skin Research and Technology* ranked 27th for highest percentage of SOC articles, with 11.18% of all articles published from 2018–2020 containing SOC‐content as compared to the mean of 16.8% across all included journals.^10^ As a non‐SOC scientific journal, *Skin Research and Technology* has a SOC percentage consistent with previous findings that revealed SOC and clinical journals had significantly higher percentages of SOC articles than their non‐SOC and basic science counterparts, respectively.^10^ The majority of SOC articles in *Skin Research and Technology* were classified as Tier 1A (70.27%), followed by Tier 2 (18.92%), Tier 1B (8.11%), and finally Tier 1E (2.70%) (Figure 1). These proportions deviated from our overall findings which revealed Tier 1B comprised the majority (61.9%) of all SOC‐related articles.^10^

**FIGURE 1:**
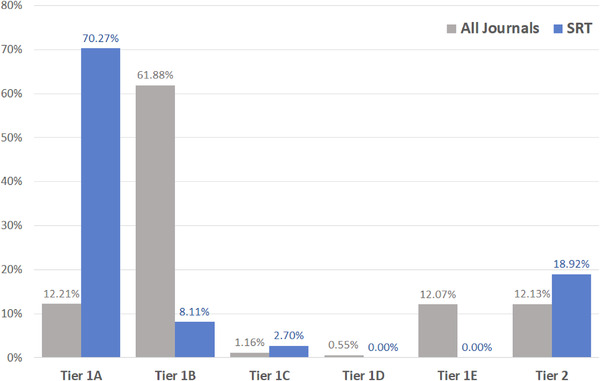
Comparing tier percentages for skin of color articles from 2018–2020, all journals and *Skin Research and Technology*

Clinically oriented scientific journals such as *Skin Research and Technology* publish foundational research upon which epidemiological and clinical studies are built. Given their potential impact on downstream research efforts and future clinical practice, it is critical to promote and measure diversity and inclusion content in these publications.^10^ As a skin imaging and bioinformatics journal, the inclusion of SOC topics and images in publications is particularly important for improving clinical education regarding SOC imaging and for improving the accuracy of computational diagnostic models and algorithms as they apply to SOC. Therefore, because *Skin Research and Technology* possesses a notable scope of influence in the field of dermatology and serves as an international resource for continuing medical education for clinicians, we encourage journal editors to evaluate submissions using our criteria for SOC content and to aim for a SOC‐relevant content of at least 17% for future issues. By enriching dermatological literature with more SOC‐related content, journals such as *Skin Research and Technology* can further diversity, equity, and inclusion within the field of dermatology.

## CONFLICT OF INTEREST

The authors have no conflict of interests to declare.
